# Headache disorder and the risk of dementia: a systematic review and meta-analysis of cohort studies

**DOI:** 10.1186/s10194-018-0925-4

**Published:** 2018-10-11

**Authors:** Jing Wang, Weihao Xu, Shasha Sun, Shengyuan Yu, Li Fan

**Affiliations:** 1 School of Medicine, Nankai University, Tianjin, 300071 China; 2 Department of Neurology, Chinese PLA General Hospital, Fuxing Road 28, Haidian District, Beijing, 100853 China; 3 Department of Geriatric Cardiology, Nanlou Division, Chinese PLA General Hospital, Beijing, 100853 China; 4 National Clinical Research Center of Geriatric Diseases, Chinese PLA General Hospital, Fuxing Road 28, Haidian District, Beijing, 100853 China

**Keywords:** Headache, Dementia, Meta-analysis

## Abstract

**Background:**

Until now, headache disorders have not been established as a risk factor for dementia. The aim of this study was to determine whether headache was associated with an increased risk of dementia.

**Methods:**

We systematically searched electronic databases, including PubMed, Embase, and Web of Science, for studies investigating the association between headache and dementia. We then conducted a meta-analysis to determine a pooled-effect estimate of the association.

**Results:**

We identified 6 studies (covering 291,549 individuals) to investigate the association between headache and the risk of all-cause dementia or Alzheimer’s disease (AD). Pooled analyses showed that any headache was associated with a 24% greater risk of all-cause dementia (relative risk [RR] = 1.24; 95% confidential interval [CI]: 1.09–1.41; *P* = 0.001), and that any headache was not statistically significantly associated with an increased risk of AD (RR = 1.47; 95% CI: 0.82–2.63; *P* = 0.192).

**Conclusions:**

Our results indicated that any headache was associated with an increased risk of all-cause dementia. However, additional studies are warranted to further confirm and understand the association.

**Electronic supplementary material:**

The online version of this article (10.1186/s10194-018-0925-4) contains supplementary material, which is available to authorized users.

## Background

Dementia is the most common neurological disease in the elderly, with devastating impact on the quality-of-life of both the patients and their family members, apart from placing a huge economic burden on the society. Over the past 20 years, researchers have been working on finding a treatment for dementia, especially that associated with Alzheimer’s disease (AD); however, results have been disappointing. Currently, there are no effective drugs that can significantly delay the progression of dementia [1]. In addition to drug treatment, researchers have also focused on the study of the risk factors for dementia in the effort that even if there is no effective treatment for the condition, the incidence thereof can still be reduced by effectively preventing and controlling the risk factors. The current identified risk factors for dementia include obesity, diabetes, hypertension, lipid metabolism disorders, coronary heart disease, and heart failure [2–5]. In addition, several studies have shown that treatment of hypertension, hyperlipidemia, and diabetes might reduce the risk of dementia [6–8].

Globally, about 45% of adults in the general population suffer from headache disorders [9]. These disorders are known to be risk factors for a variety of diseases, such as stroke, myocardial infarction and depression [10–12]. Previous studies have found migraine history to be significantly associated with cardiovascular disease and brain white-matter damage [13–15]. Non-migrainous headaches are also associated with some vascular risk factors [16, 17]. Such vascular risk factors and white-matter damage may increase the risk of dementia. Thus, headache disorders can be reasonably speculated to be associated with the increased risk of dementia. However, current evidence from longitudinal studies linking headache disorders to dementia is scarce, and study populations are often too small to detect clinically relevant associations. We therefore systematically reviewed and meta-analyzed the available longitudinal population-based evidence to determine the association of headache disorders with risk of dementia.

## Methods

### Search strategy

We conducted our systematic review and meta-analysis in accordance with the Preferred Reporting Items for Systematic Reviews and Meta-Analyses (PRISMA) statement. We systematically searched the PubMed, Embase and Web of Science databases from their inceptions to June 1, 2018 for relevant studies. Our complete search strategy is presented in Additional file 1: Table S1. Additionally, we conducted a manual search of references in the included studies and of relevant reviews to find other relevant articles. We did not apply any language restrictions.

### Selection criteria

Articles were included if they met the following criteria: [1] cohort studies; [2] report of incident dementia diagnosis as the outcome; and [3] investigation into the association of headache disorders with risk of incident all-cause dementia or of AD. Headache disorders included all types of headache. In this study, “any headache” was defined as “patient suffered from any type of headache in the past.” We chose all-cause dementia as the primary outcome measure of interest, given that the syndrome diagnosis of dementia can be defined with high consistency across studies and is less dependent on advanced diagnostic testing, which is often not feasible in large population-based studies. Nevertheless, we acknowledged the importance of the various neuropathologies underlying the clinical manifestation of dementia. We chose AD as the secondary outcome measure to provide additional insight into the association of headache disorders with dementia. If more than 1 article reported data from 1 cohort or 1 health database, we included the study with the longest follow-up or largest number of participants. Studies were excluded if they did not provide a relative-risk (RR) estimate with corresponding 95% confidence interval (CI).

Two investigators independently assessed the eligibility of the literature. First, they identified eligible articles by title and abstract; next, each of them independently read the full text of each eligible article. Discrepancies between investigators were rechecked and, if necessary, discussed with a third investigator until consensus was achieved.

### Data extraction and quality assessment

Two investigators independently extracted and summarized the relevant data of the included studies. The following information was extracted from each included study: author name, year of publication, country of study origin, population source, study design, sample size, years of follow-up, gender distribution, mean age and age range of study participants, headache type and dementia type.

We assessed the quality of the included articles using the Newcastle–Ottawa Quality Assessment Scale (NOS) [18]. Scores ranged from 0 to 9 points for cohort studies, with higher scores indicating higher study quality. We considered NOS scores of 0–3, 4–6, and ≥ 7 to indicate low, medium, and high quality, respectively.

### Statistical analysis

We extracted the adjusted RR and 95% CI from each study and used them to assess the association between headache and risk of dementia. We used random-effects models, which included assumptions about potential differences between studies, for our pooled analysis [19]. Heterogeneity of included studies was assessed by chi-square test and I-squared (I^2^) statistic. Statistical heterogeneity was considered significant when *P* < 0.10 for the χ^2^ test or when I^2^ > 50% [20]. We performed sensitivity analyses by excluding 1 study each time and re-running the analysis to verify the robustness of the overall results. We visually inspected the funnel plot to confirm publication bias. Egger’s regression test [21] and Begg’s test [22] were used to statistically assess publication bias. A 2-tailed *P*-value < 0.05 was considered statistically significant. We performed all analyses using Stata software version 12.0 (Stata Corp., College Station, Texas, US).

## Results

**Fig. 1:**
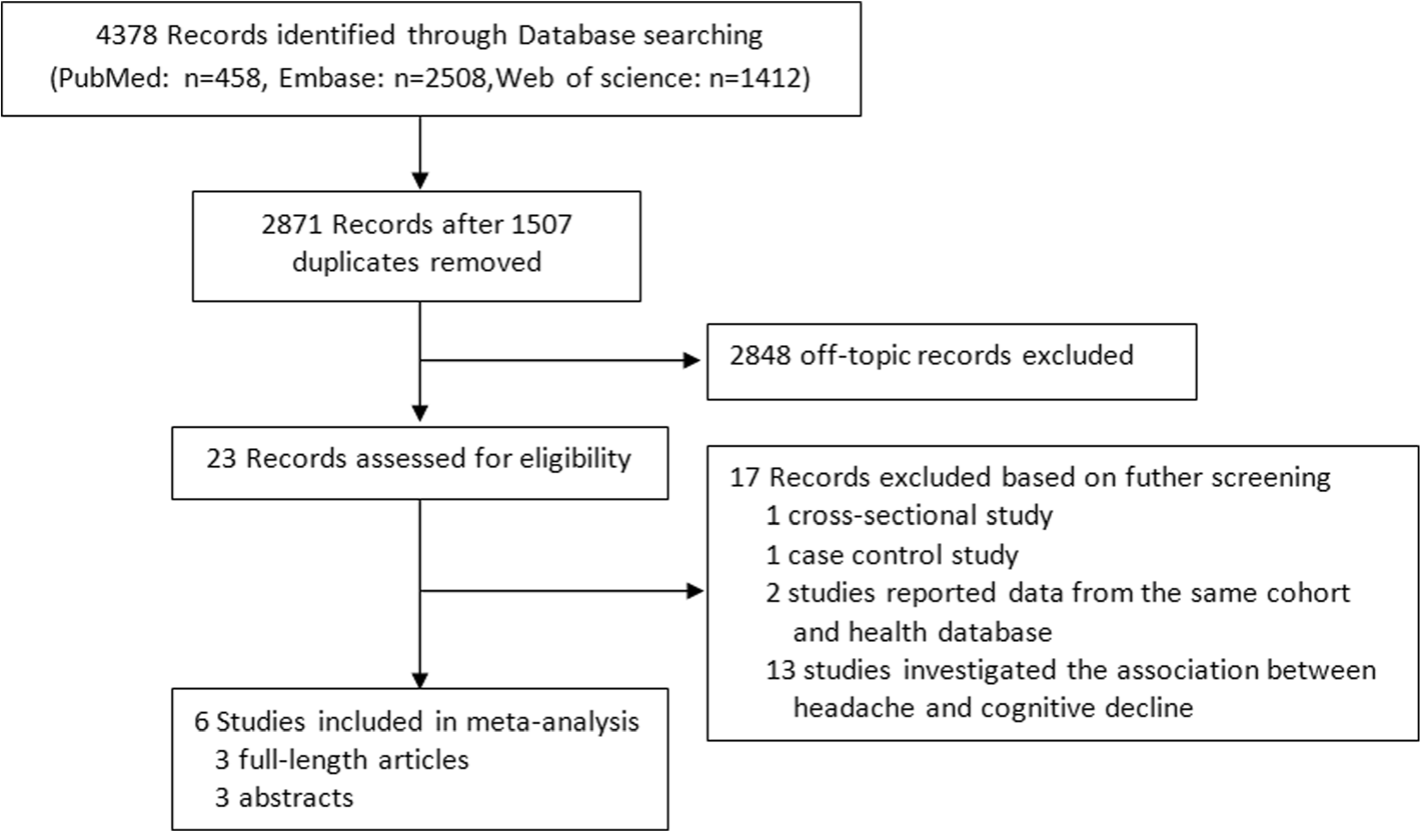
Flowchart of study identification for meta-analysis

We retrieved a total of 2871 studies from our database search. Out of those, we included 3 articles and 3 abstracts corresponding to 6 cohort studies in this meta-analysis. The study selection process is shown in Fig. 1.

### Study characteristics

Overall, we included 6 studies covering 291,549 individuals in our meta-analysis. Two studies [23, 24] were retrospective in design, while the other 4 [25–28] were prospective. Three [23–25] had sample sizes > 50,000, and the other 3 [26–28] had sample sizes < 1500. The main characteristics of the included studies are shown in Table 1.

**Table 1:** The characteristics of included cohort studies in this meta-analysis

Author year	Country	Study design	Sample size	Follow-up years	Gender	Age	Headache type	Dementia type	Confounders adjusted
Chuang 2013	China	Retrospective cohort	Total: 167,340 Migraine: 33,468 No migraine: 133,872	12 (longest)	Male and Female	42.2 (mean)	Migraine	All-cause dementia	Age, sex, diabetes, hypertension, depression, head injury and CAD
Yang 2016	China	Retrospective cohort	Total: 69540 TTH: 13,908 No TTH: 55,632	8.14 (average)	Male and Female	≥20 48.9 (mean)	TTH	All-cause dementia; VaD; AD	Age, sex, diabetes, dyslipidemia, COPD, hypertension, IHD, AF, HF, stroke, depression, head injury, Parkinson’s disease and migraine
Hagen 2013	Norway	Prospective cohort	Total: 51,859 Any headache: 21,871 No headache: 29,988	15 (average)	Male and Female	≥20 49.7 (mean)	Any headache Migraine Nonmigrainous headache	All-cause dementia; VaD; AD; Mixed dementia; VaD plus mixed dementia; Dementia with Lewy bodies; Frontotemp. dementia; Other types of dementia	Age, sex, education, total HADS score and smoking
Morton 2012	Canada	Prospective cohort	Total: 716	5 (average)	Male and Female	≥65	Migraine	All-cause dementia; VaD; AD	Age, sex, education, depression hypertension, diabetes, stroke, myocardial infarction and other heart conditions
Pavlovic 2013	USA	Prospective cohort	Total: 974 Migraine: 136 No migraine: 838	NA	Male and Female	≥70	Migraine	All-cause dementia	Age, sex, education, ethnicity, APOE-e4 carrier status, pain interference and pain intensity
Recchia 2016	Italy	Prospective cohort	Total: 1120	3.9 (average)	Male and Female	≥80	Any headache	All-cause dementia	Age, sex and education

*CAD* coronary artery disease, *TTH* tension-type headache, *VaD* vascular dementia, *AD* Alzheimer’s disease, *COPD* chronic obstructive pulmonary disease, *IHD* ischemic heart disease, *AF* atrial fibrillation, *HF* heart failure, *HADS* hospital anxiety and depression scale, *NA* not available, *APOE* apolipoprotein E

### Quality assessment

We were able to assess the quality of the 3 full-length articles only. Specific assessments with NOS scores for these 3 studies are shown in Additional file 1: Table S2.

### Any headache and risk of all-cause dementia

**Fig. 2:**
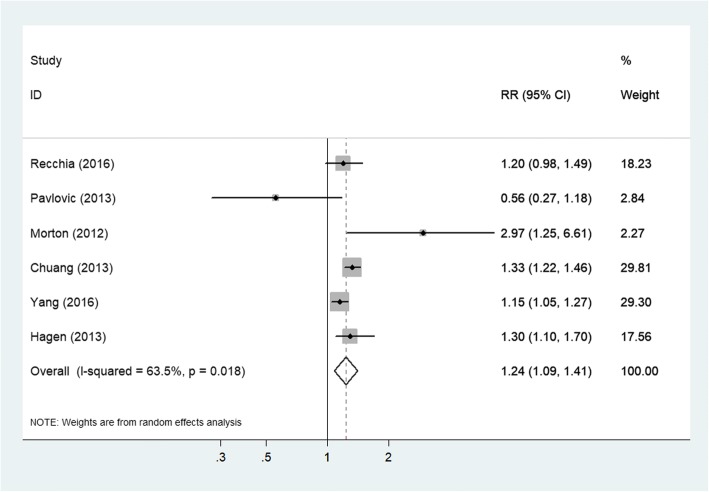
Forest plot of the association between any headache and the risk of all-cause dementia

**Fig. 3:**
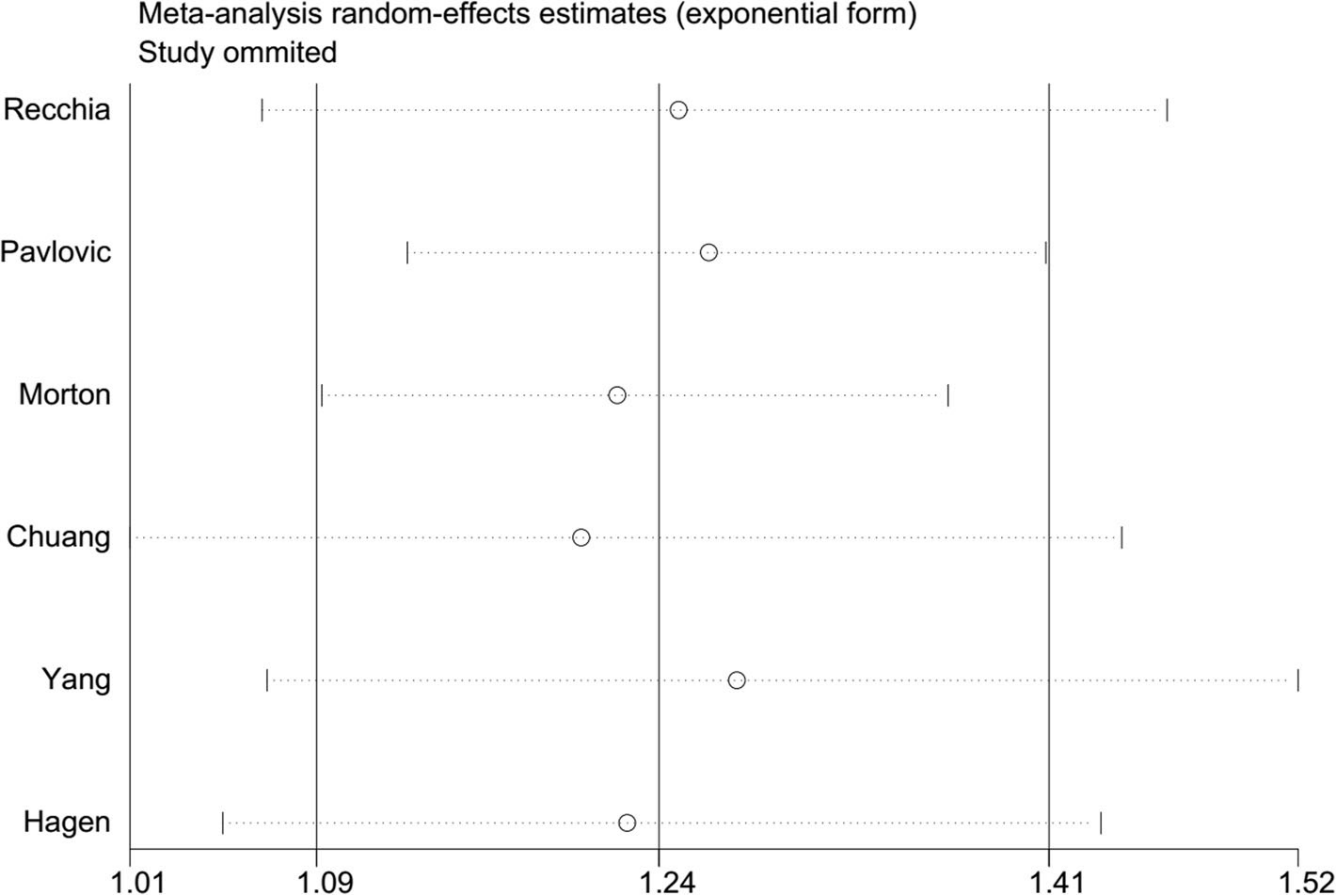
Plot of sensitivity analysis by excluding one study each time and the pooling estimate for the rest of the studies

**Fig. 4:**
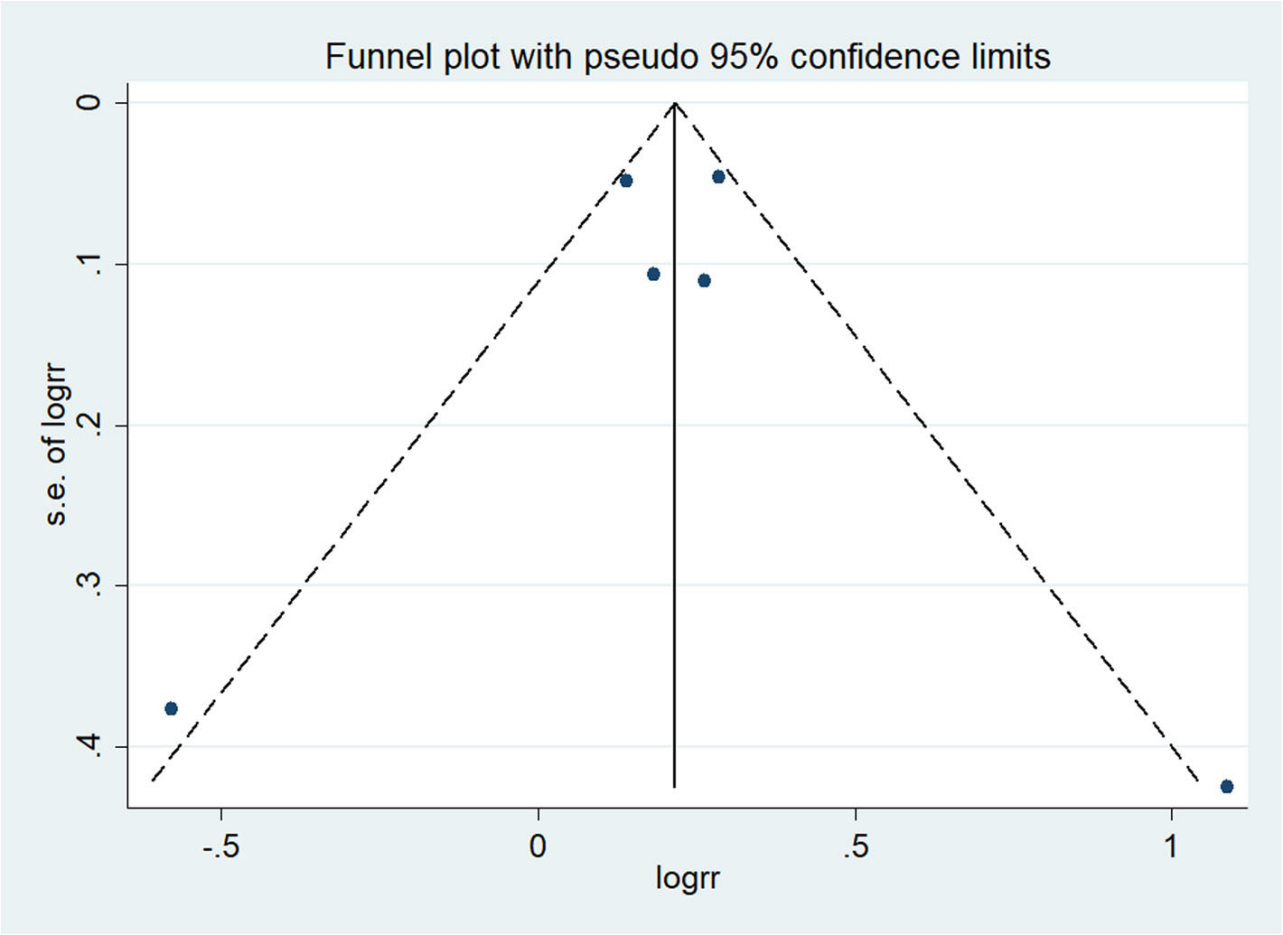
Funnel plot of log relative risk vs. standard error of log relative risks (for any headache and risk of all-cause dementia)

The 6 included studies all assessed the association between any headache and the risk of all-cause dementia. Overall, the history of any headache was associated with an increased risk of all-cause dementia (RR = 1.24; 95% CI: 1.09–1.41; *P* = 0.001; Fig. 2), but with considerable heterogeneity across studies (*I*^2^ = 63.5%; *P*_hetero_ = 0.018). Neither subgroup analysis by sample size (large or small) nor study design (prospective or retrospective) could explain the origin of this heterogeneity. Sensitivity analysis (excluding 1 trial each time and recalculating the pooled RR for the remaining studies) showed that none of the individual studies had an evident influence on the pooled-effect size (Fig. 3). The analysis verified the robustness of the results. A visual inspection of the funnel plot showed no evidence of a significant publication bias (Fig. 4). Begg’s (*P* = 0.851) and Egger’s (*P* = 0.089) regression tests likewise indicated no publication bias in this meta-analysis.

### Any headache and risk of AD

**Fig. 5:**
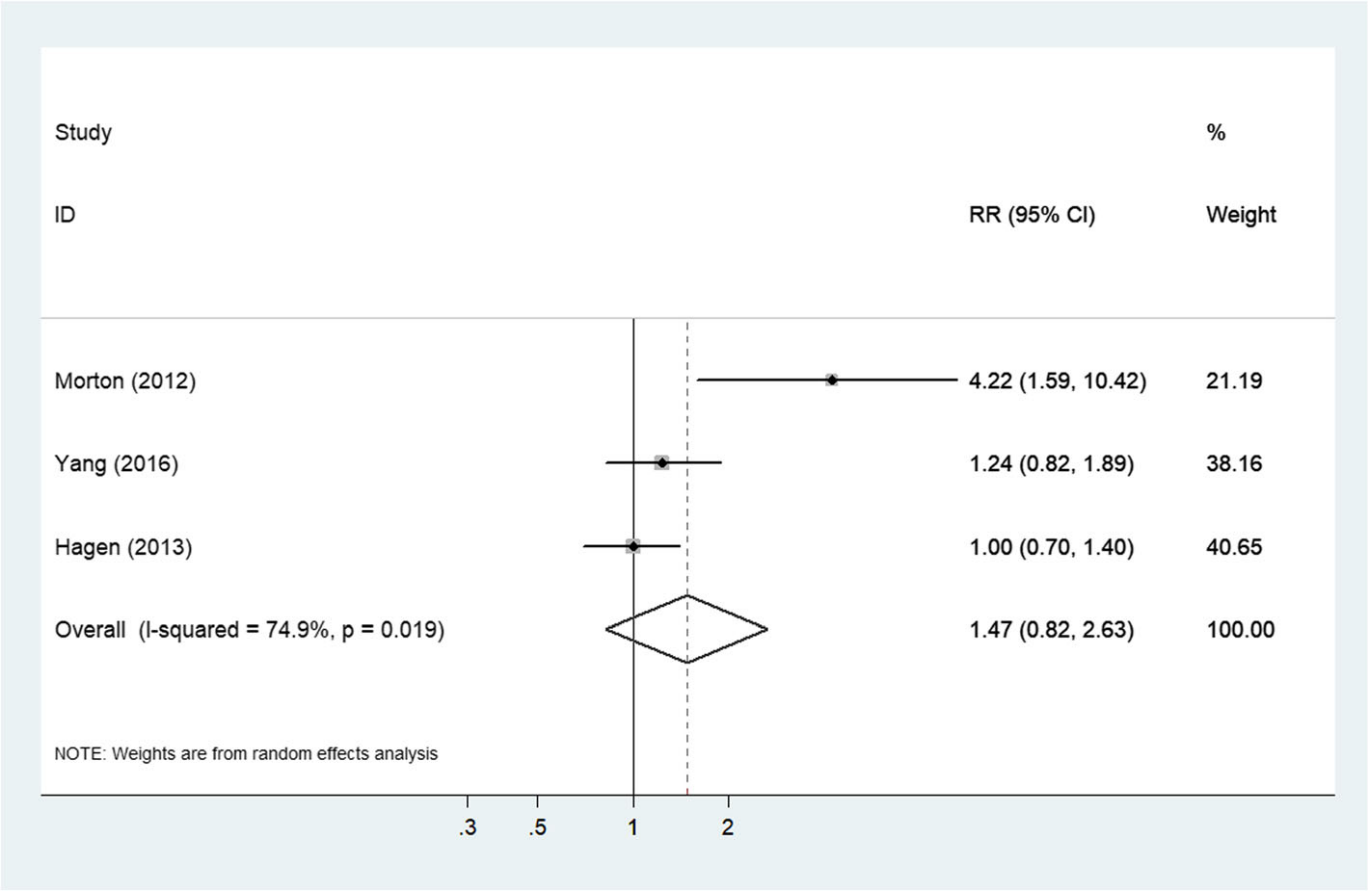
Forest plot of the association between any headache and the risk of AD

Three studies investigated the association between any headache and the risk of AD. Pooled results showed that any headache was not associated with an increased risk of AD (RR = 1.47; 95% CI: 0.82–2.63; *P* = 0.192; Fig. 5).

### Migraine and risk of dementia

Three studies reported the association between migraine and the risk of all-cause dementia. After pooling the reported effect estimates of these studies, we found an RR of 1.28 (95% CI: 0.64–2.54) for the association between history of migraine and risk of all-cause dementia. Only 1 study reported the association of migraine with the risk of AD, and its results showed that the history of migraine increased the risk of AD (RR = 4.22; 95% CI = 1.59–10.42; Additional file 1: Figure S1).

## Discussion

In this systematic review and meta-analysis of cohort studies, we compiled current evidence on the association between headache disorders and the future risk of dementia in 291,549 individuals from 6 population-based studies. We found that any headache is a potential risk indicator for all-cause dementia.

Our results were further supported by findings of 2 other large population-based studies that we did not include in this meta-analysis (reasons for exclusion are shown in Additional file 1: Table S3). Stræte Røttereng et al. used data from Nord-Trøndelag Health Surveys conducted during 1995–1997 (HUNT2) and 2006–2008 (HUNT3), and found that both any headache and non-migrainous headache were more likely to be reported in dementia patients, when compared with the control group (any headache, odds ratio [OR] = 1.24; 95% CI: 1.04–1.49 and non-migrainous headache, OR = 1.49; 95% CI: 1.24–1.80) [29]. Tzeng et al. analyzed 10 years of follow-up data from the National Health Insurance Research Database of Taiwan and suggested that patients with primary headaches had twice the normal risk of developing dementia in the future (hazard ratio [HR] = 2.06; 95% CI: 1.72–2.46) [30]. All these findings indicated that headache disorders might be a potential predictor for dementia.

It should be noted that no statistically significant result was found in the pooled analysis of the association between migraine and all-cause dementia. However, the result of this analysis was based on only three studies with two were abstracts, and as such, should be interpreted with caution. The available data on migraine and risk of AD were also unsatisfactory. Although 1 study indicated that history of migraine was associated with increased risk of AD [25], there was insufficient evidence to draw any conclusion about this association. The current available evidence on migraine and dementia were scarce but could suggest us that the possible association might exist, and highlight the need for more population-based research on this association.

The pathological association between headache disorders and dementia remains largely unknown, but several mechanisms are speculated to be involved. First, headache is a common pain disorder. A previous study found that several brain structures involved in the pain network, such as the thalamus, insula, anterior cingulate, amygdalae, and temporal cortex, undergo morphometric changes during the disease process [31]. Interestingly, these brain regions also play important roles in the memory network [32]. In addition, a previous structural–neuroimaging study of chronic headache showed that the gray-matter volume of memory network structures, including the cingulate cortex, insula, prefrontal area, and parahippocampus, decreased significantly in individuals who suffered from headache compared with those who did not [33]. These significant changes in the overlapping pain and memory networks explain the potential correlation between chronic pain and memory impairment in headache patients. Second, a previous meta-analysis found an association of white-matter hyperintensity with an increased risk of dementia [34]. Incidentally, headache patients were reported to have an increased risk of white-matter hyperintensity [35]. Therefore, subtle changes in the brain white-matter might contribute to an increased risk of dementia in headache patients. Third, depression is common, with approximate 20% of the general population experiencing a depressive episode during their lifetime [36]. An association between depression and dementia has been suggested in previous studies. Headache, especially migraine, is often comorbid with depression [37]. Specifically, previous studies found that earlier-life depression or depressive symptoms were associated with a significantly increased risk of developing dementia [38, 39]. Vascular disease, alterations in the cortisol–hippocampal pathway, increased amyloid plaque formation, inflammatory changes, and deficits in nerve growth factors or neurotrophins are predicted to be the potential biological mechanisms linking depression to dementia [40]. Thus, an increased risk of dementia in headache patients might be partly due to comorbidity with depression. Finally, stress and mental tension have been identified as predictors of headache disorder [41]. A previous cohort study found an association between psychological stress in middle-aged women and the development of dementia, especially AD [42]. The underlying mechanism remained unclear, but the hypothalamic–pituitary–adrenal axis and the effects of glucocorticoids on the brain are thought to be behind the association [43].

To the best of our knowledge, our meta-analysis was the first to summarize the currently available evidence of the association between headache and the risk of dementia, and to indicate that any headache is a risk factor for developing all-cause dementia. Sensitivity analysis verified the stabilization of the results. However, there are some limitations as well. The number of studies included was small, and the meta-analysis of the association between any headache and the risk of AD included only 3 studies. As such, the results are likely to be imprecise [44], and, consequently, the conclusions drawn from this study should be considered preliminary. In addition, the studies included show quite high heterogeneity in terms of study design, population sizes and population age range. The potential effect of the heterogeneity should be taken into account when interpreting the findings of the review. Even so, our findings are still of great significance. The association we found might aid in identifying people prone to dementia or cognitive decline. This emphasizes the need to reveal the mechanisms underlying the link between headache and dementia, which may become all the more evident while improving the quality-of-life of patients with headache disorder. The information is critical to finding new preventive and treatment strategies for dementia. It is also of crucial importance that we figure out whether treatment for headache disorder might intervene in the overlapping pathways and subsequently reduce the risk of dementia.

## Conclusions

We found that any headache was associated with an increased risk of all-cause dementia in the general population. Our results also highlighted that population-based data on the association of headache with incident dementia remains limited and that further study into the underlying mechanism of the association is warranted.

## Additional file

Additional file 1: **Table S1.** Literature search strategy. **Table S2.** Assessment of cohort studies included in this meta-analysis. **Table S3.** Excluded studies and reasons for exclusion. **Figure S1.** Forest plot of the association between history of migraine and risk of all-cause dementia. (DOCX 2302 kb)

## Abbreviations

AD: Alzheimer’s disease
CI: confidence interval
HR: hazard ratio
NOS: Newcastle–Ottawa Quality Assessment Scale
OR: odds ratio
PRISMA: Preferred Reporting Items for Systematic Reviews and Meta-Analyses
RR: relative-risk
