# Supplementary material for: Headache disorder and the risk of dementia: a systematic review and meta-analysis of cohort studies
Source: J Headache Pain. 2018 Oct 11;19(1):95. doi: 10.1186/s10194-018-0925-4 (PMC6755577; doi:10.1186/s10194-018-0925-4)
Supplement: Supplementary file 1 — Table S1. Literature search strategy. Table S2. Assessment of cohort studies included in this meta-analysis. Table S3. Excluded studies and reasons for exclusion. Figure S1. Forest plot of the association between history of migraine and risk of all-cause dementia. (DOCX 2302 kb) [file 10194_2018_925_MOESM1_ESM.docx]

Additional file 1: **Table S1** Literature Search Strategy

| Set | Search |
| --- | --- |
| 1 | Headache |
| 2 | Migraine |
| 3 | 1 OR 2 |
| 4 | Dementia |
| 5 | Alzheimer Disease |
| 6 | 4 OR 5 |
| 7 | Case report |
| 8 | Review |
| 9 | Meta-analysis |
| 10 | 7 OR 8 OR 9 |
| 11 | (3 AND 6) NOT 10 |

**Table S2.** Assessment of cohort studies included in this meta-analysis

| First author, year | Representativeness  of the exposed cohort | Selection of the non- exposed cohort | Ascertainment of exposure | Demonstration that outcome of interest was not present at start of study | Comparability | Assessment of the outcome | Was follow-up long enough for outcomes to occur | Adequacy of follow up of cohorts | Total  score |
| --- | --- | --- | --- | --- | --- | --- | --- | --- | --- |
| Yang 2016 | * | * | * | * | ** | * | * | - | 8 stars |
| Hagen 2013 | * | * | * | * | ** | * | * | - | 8 stars |
| Chuang 2013 | * | * | * | * | ** | * | * | - | 8 stars |

** Quality criterion completely satisfied; * quality criterion satisfied;

**Table S3.** Excluded studies and reasons for exclusion

| Excluded studies | Reasons for exclusion |
| --- | --- |
| [Stræte Røttereng](http://106.39.55.6/s/gov/nih/nlm/ncbi/www/G.https/pubmed/?term=Str%C3%A6te%20R%C3%B8ttereng%20AK%5BAuthor%5D&cauthor=true&cauthor_uid=26471177)  2015 | Although this study examined the association between headache and dementia, the authors did not focus on future risk of dementia in patients with or without headache history, but only reported those dementia patients were more likely to have headache history. |
| Tzeng  2016 | Patients included in this study were also included in Yang’ study (Ref 23) and Chuang’ study (Ref 24). And the two other studies included more headache patients than this study. |


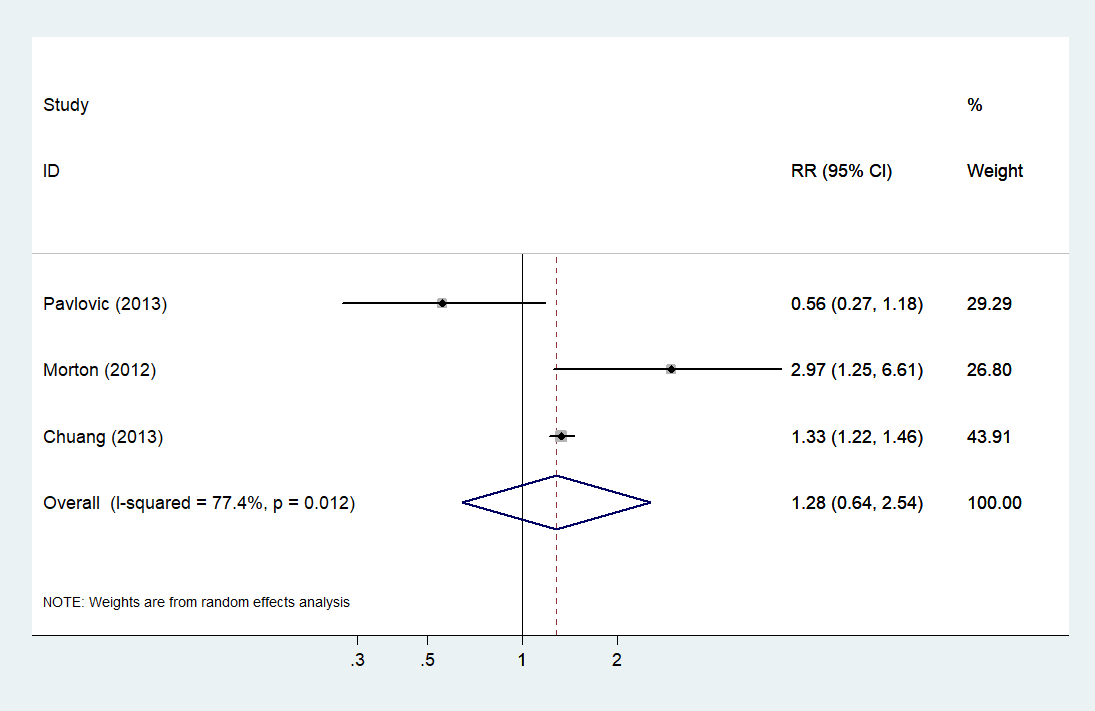


**Figure S1.** Forest plot of the association between history of migraine and risk of all-cause dementia
